# Guidelines to use tomato in experiments with a controlled environment

**DOI:** 10.3389/fpls.2014.00625

**Published:** 2014-11-18

**Authors:** Dietmar Schwarz, Andrew J. Thompson, Hans-Peter Kläring

**Affiliations:** ^1^Department of Plant Nutrition, Leibniz Institute for Vegetable and Ornamental CropsGroßbeeren, Germany; ^2^Reader in Molecular Plant Science, School of Energy, Environment and Agrifood, Cranfield UniversityCranfield, UK; ^3^Department of Modelling and Knowledge Transfer, Leibniz Institute for Vegetable and Ornamental CropsGroßbeeren, Germany

**Keywords:** CO_2_concentration, cultivation techniques, genetic variation, light, nutrition, *Solanum lycopersicum*, temperature, transpiration

## Abstract

Domesticated tomato (*Solanum lycopersicum*) is the most important horticultural crop worldwide. Low polymorphism at the DNA level conflicts with the wealth of morphological variation. Fruits vary widely in size, shape, and color. In contrast, genetic variation between the 16 wild relatives is tremendous. Several large seed banks provide tomato germplasm for both domesticated and wild accessions of tomato. Recently, the genomes of the inbred cultivar “Heinz 1706” (≈900 Mb), and *S. pimpinellifolium* (739 Mb) were sequenced. Genomic markers and genome re-sequencing data are available for >150 cultivars and accessions. Transformation of tomato is relatively easy and T-DNA insertion line collections are available. Tomato is widely used as a model crop for fruit development but also for diverse physiological, cellular, biochemical, molecular, and genetic studies. It can be easily grown in greenhouses or growth chambers. Plants grow, flower, and develop fruits well at daily light lengths between 8 and 16 h. The required daily light integral of an experiment depends on growth stage and temperature investigated. Temperature must be 10–35°C, relative humidity 30–90%, and, CO_2_ concentration 200–1500 μmol mol^−1^. Temperature determines the speed of the phenological development while daily light integral and CO_2_ concentration affect photosynthesis and biomass production. Seed to seed cultivation takes 100 days at 20°C and can be shortened or delayed by temperature. Tomato may be cultivated in soil, substrates, or aeroponically without any substrate. Root volume, and water uptake requirements are primarily determined by transpiration demands of the plants. Many nutrient supply recipes and strategies are available to ensure sufficient supply as well as specific nutrient deficits/surplus. Using appropriate cultivation techniques makes tomato a convenient model plant for researchers, even for beginners.

## Tomato for experimental use

### Introduction

The goal of this paper was to prepare a guide for tomato cultivation, particularly for beginners, and experimenters with not much experience with tomato. Actually, many protocols and helpful guides are available that explain and summarize how to grow tomato (See General Consideration). However, they are prepared rather for farmers and horticulturists and thus not that suitable for experimental purposes. Moreover, some of them are difficult to find and receive by libraries. For experimental purposes, many scientific papers describe their approaches in the Materials and Method Section. Sometimes, these protocols are difficult to follow without having the same experience, they are very specific and not suitable for the actual purpose, or they are too general and therefore, also leave open many specific problems (Poorter et al., 2012). Therefore, this guide aims to summarize and simplify the available knowledge considering the experimental conditions in climate chambers and greenhouses. Here, the authors feed upon many years of experience with tomato experiments which include many mistakes that readers can hopefully avoid. The authors would very much appreciate if readers and users respond to the authors with their own experience and help to update and improve this protocol for future use.

### Background, general use of tomato for experimental purposes

Cultivated tomato belong to the Solanaceae family and the genus *Solanum*. They are not only the most popular vegetable crop but also the most cultivated vegetable worldwide (4.7 Million ha). Moreover, tomato is one of the most studied fleshy fruits because it is easy to grow, often used to explore its characteristics, or used as a model plant. Recently the genome was published (Sato et al., 2012) and many insertion line collections and mutants exist, making the use of tomato as an experimental plant even more promising.

The offer in different tomato is huge (Van der Knaap et al., 2013) and thus, the selection of suitable plants for experimental aims might be difficult. The experimenter needs to decide and can consider in favor of many different selection criteria. An overview can be found in the UPOV guidelines (2001). Here, only an excerpt is given, such as:

- Growth habit is predominantly controlled by the *self-pruning* (*sp*) locus.
  - Determinate (bush type) carries the recessive mutant allele *sp* (http://tgc.ifas.ufl.edu/genelist.htm). It produces a limited number of trusses, depending on plant, and climate conditions. The number of leaves or internodes between inflorescences varies from one to three. In the terminal truss the stem ends with an inflorescence and no lateral shoots are produced. Determinate plants are very suitable for experiments with limited space and where nevertheless fruits are wanted. e.g., “Micro Tom” is considered to be the world's smallest tomato, with plants only 13–20 cm tall (Martí et al., 2006; Carvalho et al., 2011; Okabe et al., 2011).
  - Indeterminate (standard or vine type, tall): carries the dominant wild type allele (*sp*^+^). Three leaves or internodes are generally observed between inflorescences. Each sympodial segment produces three buds: the terminal bud is transformed into a flowering bud; one of the two axillary buds is transformed into a lateral shoot which produces the next three buds and carries on the prolongation of stem. Plants of this type grow with the continuous repeat of this growth pattern, known as sympodial growth (Heuvelink, 1995a).
  - Semi-determinate (intermediate type): this phenotype does not have three leaves or internodes consistently between inflorescences, and shows semi-determinate growth, e.g., with the termination of the stem prolongation above ninth inflorescence.
- Fruit traits. From 24 different traits (UPOV guidelines, 2001), the following are selected. Possible representative cultivars for the traits are given in the UPOV guidelines:
  - Size/weight/shape (Table 1).
  - Size and shape variation is controlled by key loci (Tanksley, 2004). The specification is very diverse and includes five classes in size from very small to very large and seven classes in shape (longitudinal section) varying from near-spherical to more or less flattened, to pear- or cylindrically-shaped (Van der Knaap et al., 2013). A cross section reveals if the fruit is a round type or not.
  - Number of locules. It varies greatly in commercial cultivars.
    - Two (e.g., cherry, plum or some pear types),
    - two or three,
    - three or four,
    - four, five, or six,
    - more than six (e.g., large beef tomato).
  - Color. The fruit color is determined by skin and flesh color. Both skin and flesh color vary between cream (colorless) to orange to brownish, depending on the amount and type of carotenoid, and flavonoid pigments, and whether chlorophyll persists in the flesh (Lindhout, 2005). In general, the darker red the fruit the more lycopene it contains. Red is still the major fruit color for most of the commonly grown varieties. Color of the skin is controlled by the *y* locus, where the *y* mutant fails to accumulate a yellow-colored flavonoid naringenin chalcone in the epidermis, giving fruit a pink color when the flesh is red (Ballester et al., 2010).
  - Purpose of main use, for
    - fresh market or garden
    - processing
    - pot plant
- Disease resistance (Grandillo et al., 2013). The resistance of a cultivar can have different levels, e.g., high or intermediate. The information is released by the breeder following the international standardized nomenclature of codes for pest organisms International Seed Federation (2013). Modern cultivars will tend to have resistance to multiple key diseases, whereas heirloom varieties may not. Therefore, extra care should be taken in controlling plant diseases when selecting heirloom cultivars. A common notation is “VFNT,” indicating resistance to *Verticillium* wilt, *Fusarium* wilt, nematodes, and tobacco mosaic virus. Breeding companies use molecular markers to stack-up known disease resistance alleles into their elite germplasm. Methods to tests resistance against the main tomato pests and diseases and an overview on susceptible and resistant varieties are summarized in the UPOV guidelines (2001).

**Table 1:** **Tomato fruit size, mass, and diameter (adapted from UPOV guidelines, 2001 and Costa and Heuvelink, 2005)**.

**Fruit size**	**Type**	**Mass (g)**	**Diameter (mm)^a^**
Very small	Cherry	1–20	<25
Small	Cocktail	20–70	25–47
Medium	Intermediate	70–100	47–67
Large	Intermediate	100–180	67–88
Very large	Beef	>180	>88

a *Depends on shape*.

## Plant material

### Selection of species, cultivars, or mutants

The number of different tomatoes available is overwhelming. However, following the classification of plants there are only 13 different species (Table 2) (Peralta et al., 2008; Bauchet and Causse, 2012; Van der Knaap et al., 2013). Within these species many botanical varieties are regulated by the *International Code of Nomenclature for algae, fungi, and plants* (McNeill et al., 2012). The cultivated varieties (=cultivars) are listed and regulated by the *International Code of Nomenclature for Cultivated Plants* (Brickell et al., 2009). Cultivars may be also interspecific that include DNA from a wild species, developed following a cross between a wild species and cultivated tomato. In addition, many genotypes and mutants are available. Therefore, the selection of a suitable tomato necessary before the start of an experiment is often very difficult. A helpful overview can be found at the web page of the Tomato Genetic Resource Center (TGRC)^1^, in the UPOV guidelines (2001), or in the book of Liedl et al. (2013). The database of the TGRC contains actually more than 1000 types of domesticated and more than 1200 accessions of wild tomato.

**Table 2:** **Tomato species of the genus Solanum, Section Lycopersicon, and the former Lycopersicon synonyms**.

**Genus: Solanum**	**Lycopersicon synonyms**	**Cross compatible**
*S. lycopersicum* L.	*L. esculentum* Miller	Domesticated species
*S. cheesmaniae* (Riley) Fosberg	*L. cheesmaniae* Riley	Fully
*S. galapagense, S. Darwin and Peralta*	*L. cheesmaniae, L. Riley* (forma or var. minor)	Fully
*S. pimpinellifolium* B. Juss.	*L. pimpinellifolium* (B. Juss.) Miller	Fully
*S. chmielewski*^a^	*L. chmielewski* C. M. Rick, Kesicki, Foboes and M. Holle	Difficult^b^
*S. habrochaites* S. Knapp and D. M. Spooner	*L. hirsutum*	Difficult
*S. neorickii*^a^	*L. parviflorum* C. M. Rick, Kesicki, Foboes and M. Holle	Difficult
*S. pennellii* Corell	*L. pennellii* (Correll) D'Arcy	Difficult
*S. chilense* (Dunal) Reiche	*L. chilense* Dunal	Very difficult^b^
*S. corneliomuelleri J. F. Machr*.	*L. peruvianum* (L.) Miller *L. glandulosum* C. F. Mull.	Very difficult
*S. huaylasense* Peralta	*L. peruvianum* (L.) Miller	Very difficult
*S. peruvianum* L.	*L. peruvianum* (L.) Miller	Very difficult
*S. arcanum* Peralta	*L. peruvianum* (L.) Miller	Very difficult

*Cross compatibility with the domesticated S. lycopersicum (Peralta et al., 2008; Grandillo et al., 2013 see also at TDRC)*.

a *(C. M. Rick, Kesicki, Foboes, and M. Holle) D. M. Spooner, G. J. Anderson, and R. K. Jansen*.

b *(Very) difficult means that crossings need special attention and courses of action*.

The polymorphism at the DNA level within the species *Solanum lycopersicum* is rather small but the differences between species are large. Morphological variation, such as growth habit, color, etc. (see Background, General use of Tomato for Experimental Purposes), is also huge within *S. lycopersicum*. An experiment related to commercial conditions should utilize commercial cultivars with the appropriate growth habit and morphology. Each year breeding companies release plenty of new cultivars to the market, making the cultivar selection even more difficult. If the experimenter prepares a set of trials even over several years, it is recommended to select a common representative cultivar and to stock sufficient seeds for the work. Breeders may help with the selection but be careful when asking breeding companies since they always recommend their own assortment.

### Genetic diversity

Cultivated tomato is diploid and self-compatible, although some wild-species are self-incompatible. Extended physical and genetic maps with lots of markers are available (http://solgenomics.net) and numerous interesting quantitative trait loci (QTLs) are mapped. Recently, the genomes of the inbred cultivar “Heinz 1706” (≈900 Mb) and *S. pimpinellifolium* (739 Mb) were sequenced (Sato et al., 2012), and 150 accession were “re-sequenced,” i.e., next generation sequence data were mapped to the Heinz 1706 reference to define many thousands of DNA polymorphisms between these accessions (http://www.tomatogenome.net/). Since the advances in next generation sequencing technology it has become possible to define the thousands of genetic polymorphisms between any two cultivars for only a few thousand Euros or Dollars, making genetic analysis easy and cheap. A wealth of genomic resources are now available, including DNA polymorphisms (e.g., Causse et al., 2013) gene expression data (e.g., Koenig et al., 2013) and emerging whole genome assemblies of wild species to complement the Heinz 1706 reference sequence (Table 3.1).

Table 3**Tomato Genomic Resources**.

**Table:** 

**3.1. Databases and resource centers**		
**Name**	**Location**	**Web address**
Sol Genomics Network	Sol Genomics Network, Boyce Thompson Institute for Plant Research, Room 221, Tower Road, Ithaca, NY 14853, USA	http://solgenomics.net/
150 Tomato Genome Resequencing project	Wageningen UR, Droevendaalsesteeg 4, 6708 PB Wageningen, The Netherlands	http://www.tomatogenome.net/
Tomato Genomic Resources Database	Dr. Debasis Chattopadhyay, National Institute of Plant Genome Research, Aruna Asaf Ali Marg, PO Box No. 10531, New Delhi - 110 067, India	http://59.163.192.91/tomato2/
Tomato Functional Genomics Database	Boyce Thompson Institute for Plant Research, Cornell University	http://ted.bti.cornell.edu/
Tomato Epigenome Database	Boyce Thompson Institute for Plant Research, Cornell University, USA	http://ted.bti.cornell.edu/epigenome/
Solanaceae Coordinated Agricultural Project	Michigan State University, East Lansing, MI 48824, USA	http://solcap.msu.edu/index.shtml

**Table:** 

**3.2. Key germplasm collections, TILLING resources and mutant collections**		
**Name**	**Location**	**Web address**
**KEY GERMPLASM COLLECTIONS**		
C. M. Rick Tomato Genetics Resource Centre	University of California, Davis	http://tgrc.ucdavis.edu/
The AVRDC Vegetable Genetic Resources Information System (AVGRIS)	Genetic Resources and Seed Unit AVRDC The World Vegetable Center, PO Box 42, Shanhua, Tainan, Taiwan	http://203.64.245.173/ or http://avrdc.org/
United States Department of Agriculture, Agricultural Research Service, Tomato Collection	630 West North Street, Geneva, NY 14456, USA	http://www.ars.usda.gov/Aboutus/docs.htm?docid=6452
**TILLING RESOURCES (REVERSE GENETICS) AND MUTANT COLLECTIONS (FORWARD GENETICS)**		
Plant Genomics Research, Unité de Recherche en Génomique Végétale	INRA/CNRS – URGV, 2, INRA/CNRS – URGV, 2, rue Gaston Crémieux, CP5708, 91057 Evry cedex, France	http://www-urgv.versailles.inra.fr/tilling/tomato.htm
The University of California, Davis, Genome Center TILLING laboratory	Comai Lab – TILLING, 451 Health Sciences Drive, Davis, CA 95616-8816, USA	http://tilling.ucdavis.edu/index.php/Tomato_Tilling
“Genes that make Tomatoes” Laboratory of Dani Zamir	The Robert H. Smith Institute of Plant Sciences and Genetics in Agriculture, The Hebrew University of Jerusalem, Israel	http://zamir.sgn.cornell.edu/mutants/
TomaToma: Tomato mutants archive	NBRP tomato, Gene Research Center, University of Tsukuba, 1-1-1 Tennodai, Tsukuba, Ibaraki 305-8572, Japan	http://tomatoma.nbrp.jp/
Dr. Lazaro Peres, Laboratory of Hormonal Control of Plant Development – Collection of Micro-Tom mutants	Universidade de São Paulo (USP), Escola Superior de Agricultura “Luiz de Queiroz” (ESALQ), Departamento de Ciências Biológicas (LCB), Piracicaba, SP - Brazil	http://www.esalq.usp.br/tomato

*A full list of germplasm collections, including tomato is available from FAO: http://www.fao.org/docrep/013/i1500e/i1500e00.htm (The Second Report on The State of the World's Plant Genetic Resources for Food and Agriculture)*.

Crossing is the key step for genetic analyses and breeding of tomato. Since tomato are self-pollinating plants, to cross pollinate, emasculation (removal of immature anthers from the female parent) is essential. All wild tomato species can be crossed with cultivated tomato; this is useful because wild tomato are a great source of desirable traits. Thus, within the genus *Solanum*, some, such as *S. pimpinellifolium*, are closely related to domesticated tomato cultivars and are fully cross compatible; others, such as *S. peruvianum*, are more distantly related (Table 2). Some wild species have been effectively crossed with the cultivated tomato *S. lycopersicum* and successfully used as donors of genes conferring resistance to pathogenic fungi and insects (*S. habrochaites* and *S. peruvianum*), for fruit quality improvement (*S. chmielewskii*), and for adaptation to adverse environments (*S. cheesmaniae*) (Esquinas-Alcazar, 2005; Grandillo et al., 2013).

Many commercial tomato are F_1_ hybrids. The seeds for them were produced by crossing two inbred tomato parents resulting in genetically identical F_1_ seeds with hybrid vigor. The parental lines are closely guarded by seed companies so that only they can produce the F_1_ seeds.

### Transformation, insertion, isogenic line, and mutant collections

Tomato is simple to transform by *Agrobacterium tumefaciens*. It can be routinely carried out and numerous institutes have service labs to do it. It is recommended to cooperate with such institutes. Transformation is used for functional analyses of genes where natural mutants are not available. Transgenic lines for numerous genes might be available from authors who have published the production of these lines. If not and transformation must be carried out oneself, the host cultivar must be carefully selected because the competence to transformation varies greatly between cultivars. The cvs “Moneymaker,” “Ailsa Craig,” and “Micro-Tom” are known to be simple to transform. Attempts have been made to identify genes by insertional mutagenesis (e.g., Atares et al., 2011), but a central collection does not exist.

Many mutants and isogenic lines have been identified that are involved in all kinds of tomato characteristics. Suitable mutants to investigate the relationship between gene identity and gene function can be obtained from a number of germplasm collections (Table 3.2) or can be requested directly from specific labs responsible for the discovery and publication of new mutants. In addition, there are now several TILLING resources available for tomato (Table 3.2); TILLING allows mutations to be found potentially in any target gene (Okabe et al., 2011). The use of the Micro-Tom genetic background in which the small size is conferred by three genetic loci, allows some experiments, where plant size is critical, to be carried out with minimal resources, and with a more rapid cycling through generations (Carvalho et al., 2011).

### Seed characteristics and propagation

The mean seed weight is about 3.3 mg. Seeds can be stored at room temperature for a few years. To be stored for longer time periods conditions have to be dry (relative humidity < 20%) and cool (−20°C). Thus, it is possible to have even after decades seeds with a high germination rate.

The number of seeds per fruit in tomato is very variable according to the genotype. Production is simple and one plant can provide large quantities of seeds, often each fruit contains more than 100 seeds. Except hybrids, seeds of many other genotypes can be easily self-propagated in a greenhouse or growth chamber as described in Cultivation. To ensure large seed production, vibrate flowering trusses for pollination. Since many tomato are self-pollinating and to avoid cross pollination, one flower or a whole flower truss should be covered with a paper bag. As soon as the fruits appear the paper bag can be removed. Extraction of mature seeds from the fruits harvested results in a gelatinous seed mass that needs to be cleaned by application of 0.7% HCl or 1% H_2_SO_4_ for 1–3 h. The addition of pectolase (1 g/L, available from brewing shops) to a 1% concentrated HCl solution and incubation of the seed-gel (v/v) overnight at ambient temperature (≈20°C) improves the extraction.

### Seedling production

Seedlings can be self-produced or ordered as transplants from a seedling company. Seeds germinate uniformly on average after 4 days when they are kept at 25–27°C. Some of the new cultivars and rootstocks are primed and germinate earlier, after 2–3 days. Wild types, other species than *S. lycopersicum*, or mutants often need more time for germination or germinate poorly. Germination can be improved by repeated treatment with sodium hypochlorite (http://tgrc.ucdavis.edu/seed_germ.aspx). To prevent fungal growth and transmission of seed-borne viral diseases, seeds should routinely be surface sterilized in 1% Na-hypochlorite solution for 20–30 min. Thereafter, they need to be rinsed with distilled water several times.

The self-production of seedlings can be carried out following different methods:

- About 100–200 seeds can be broadcasted or sown in rows as even as possible in a plastic tray (about 0.4 × 0.2 m) containing a compacted and well watered substrate (e.g., peat or coarse sand, see Table 4) that should be the same as used in the main experiment. The seeds need to be covered with 5 mm of fine-grade growing medium and then with paper or a glass plate to prevent drying out. However, it is also common to use a coarse sand (gravel size of about 0.1–2 mm), particularly for experiments in hydroponic systems without any substrate and when the root is on focus. After germination the special medium wanted for the experiment can be selected. Sand makes it easy to have a plant root system without any substrate. Since sand has no optimal composition of the air, water and solid phase it takes longer to get the seedlings germinated than using an e.g., organic or horticultural substrate. Plants are less disturbed during transplanting since relatively clean roots can be easily removed from the sand.
- About 20 seeds can be sown on filter paper covering the bottom of a Petri dish with a size of 150 × 15 mm (Di Salvatore et al., 2008). The paper is wetted with 5–10 ml of sterile demineralized water, depending on the filter paper used. Under dry conditions thicker paper or two layers should be used. The Petri dishes were then wrapped with Parafilm™ and placed in a germination chamber.
- Instead of filter paper, seeds can also be sown in a growing media, such as agar 0.6% (w/v), filled in a Petri dish, vessel, or other container (Di Salvatore et al., 2008). Suitable glassware for the germination of 20 seeds are e.g., vessels of 120 × 40 mm where the seeds are placed flat. Vessels should be stored about 4 days (or for a different germination time as known from the cultivar) under dark conditions at 25°C. When roots are longer than 5 mm they can be transferred.

**Table 4:** **Important possible growing media (modified after Ter Berg, 2000)**.

**Substrate**		**Raw material**	**Characteristics**				
			**AV^a^**	**WV^b^**	**OM^c^**	**Density^d^**	**RD^e^**
			**m^3^ m^−3^**	**m^3^ m^−3^**	**g g^−1^**	**kg m^−3^**	**kg m^−3^**
None	NFT	Nutrient film technique	–	0.99	0	–	–
	DFT	Deep flow technique	–	0.99	0	–	–
	Aeroponic	Foggy nutrient solution	0.99	–	0	–	–
Inert	Sand, gravel, or mixes	Granular material of fine divided rock and mineral particles	0.20 depending on particle size				
	Glass wool	Melted silica sand (1200°C)	^f^				
	Foam	Phenolic foam granules based on mineral oil	^f^				
	Rock wool	Melted basalt and lime stone after supply of coke (1600°C)	0.04	0.94	0.02	1900	49
	Lava	Brocken, sieved vulcanic stone	^f^				
	Pumice	Porose, air filled vulcanic stone	0.26	0.57	0.03	2000	431
	Perlite	Morphous volcanic glass	0.52	0.44	0.01	900	105
	Vermiculit	Hydrous silicate mineral	^f^	0.50		2500	70–100
	Vleece	Polyester, etc.	^g^				
	Clay granules	Decompressed clay (1100°C)			0	1870	489
	Polyurethan	Mix of mineral oil and di-isocyanat	0.35	0.60	0.99	1190	78
Organic	Wood fiber	Pressed wood mill waste	0.62	0.20	0.98	1440	87
	Saw dust	Different origins	^g^				
	Peat		0.14	0.79	0.96	1580	113
	Coco fiber	Different origins	0.55	0.40	0.98		0.77
	Rice husks	Different origins	^g^				
	Composts	Different origins	^g^				

*Suitability depends on availability, growing conditions, and experimental goal (Ter Berg, 2000)*.

a Air volume;

b water volume;

c organic portion;

d density of the solid matter;

e bulk density;

f physical and chemical characteristics insufficiently known;

g *depending on origin, produce characteristics may differ significantly*.

If researchers want to do long-term experiments or grow indeterminate tomato for yield it is advisable to use and to start the experiment with transplants. They should be planted after 7–12 leaves have formed, the growing point changed from vegetative to reproductive, and a cluster of flower buds appeared.

In any case, sow more seed than needed that uniform plants can be selected for the experiment. The minimum recommended is 20% more than the desired amount. The older the seeds and the higher the morphological variation within the genotype, the larger must be the number of seeds needed for germination to select sufficient uniform plants later on.

## Cultivation

### General consideration

Here only a rough overview summarizing the most important steps and characteristics in growing tomato can be mentioned. Several helpful guides, instruction manuals, textbooks, and publications may assist in more detail and are listed as follows:

- World vegetables (Rubatzky and Yamaguchi, 1996).
- Growing greenhouse tomatoes in soil and in soilless media (Papadopoulos, 1991).
- Growing greenhouse vegetables (Anonymous, 2000).
- Tomatoes (Heuvelink, 2005).
- Tomatoes: Cultivation, varieties and nutrition (Higashide, 2013).
- Tomato plant culture in the field, greenhouse, and home garden (Benton-Jones, 2008).
- Guidelines for the conduct of tests for distinctness, uniformity, and stability (UPOV guidelines, 2001).
- Integrated pest management for tomato (Strand, 1998).

In addition, several companies (e.g., for fertilizers or pesticides) and extension services in many countries offer guides how to grow tomato successfully. They can be found printed in the language of the country or even in English either as small leaflets/textbooks or directly downloadable via the internet.

To compare phenological growth stages within the solanaceae species and even with other species a uniform coding system can be used (Feller, 2001). Following the BBCH-monograph tomato is described with nine phenological growth stages, such as germination, leaf development, formation of side shoots, inflorescence emergence, flowering, development of fruit, ripening of fruit and seed, and senescence.

### Growing systems and growing media

Growing seedlings and vegetative plants, respectively, in short-term experiments for several days or up to 6 weeks is simple. Systems are offered, such as:

- Lab-vessels with a substrate, e.g., agar (small seedlings).
- Pots with growing media (see Table 4, Ter Berg, 2000). Depending on the tomato size wanted at the end of the experiment, pot diameters should be selected between 8 and 16 cm.
- Glass containers or containers made of other materials filled with nutrient solution (see Nutrients and Recipes). In our experiments, best practice have been containers with a two liter volume since they can be easily cleaned and sterilized. Containers can be wrapped with an aluminum or a black-and-white plastic film to avoid light transmission to the roots and to prevent algae growth in the nutrient solution. To secure that roots are properly submerged in the nutrient solution and that shoots are above the surface, a foam plate floating on the surface of the nutrient solution can be used.

Growing fruits and producing seeds in long-term experiments requires advanced standards compared with just cultivating vegetative tomato. Here, we can learn from farmers since they developed and used uncountable cultivation systems during the last 30 years.

The following systems are simple to handle and therefore, recommended. Moreover, they can be adapted to different available conditions and the growing medium can be substituted depending on the available material or other criteria.

- Containers. The size selected for a long-term experiment should have a minimum of 12 liters. To increase pot size with the increase of the plant size is possible if wanted. In each case the pot should gather all roots and assure sufficient air and water distribution, otherwise, root restriction can become a factor that induces additional effects and produces experimental artifacts. All materials and types offered for vessels are possible and can be used, such as pots, buckets, containers. Attention should be paid to very new plastic containers since they might release toxic gases. Pots with a light color or wrapped with an aluminum film should be preferred, since temperature in the substrate and thus for the roots may increase when pots with a dark/black color are used in experiments at high radiation, particularly in climate chambers. For repeated use and work with tomato pathogens stainless steel containers are recommended since they are easy to sterilize.
- Hydroponic systems either with or without recirculation. These systems have gullies that can be produced in all sizes and materials. Even a wooden frame covered with plastic foil is possible. It can have a supply tank that serves also as a drain tank and the nutrient solution is moving through the gully by gravity, or optionally both, a supply and drain tank. The system can be closed or open and the drained solution can be discarded. To prevent too much waste, a nutrient solution surplus of max. 30% above plant demands is recommended in open systems.

Both examples can be handled either with or without any growing medium. Growing media can be the soil in a greenhouse bed or any one of the many different substrates available on the market (see Table 4; Ter Berg, 2000). The substrates can be subdivided either into inert or organic materials. Both have varying characteristics that need to be considered since they affect both the water holding and the ion exchange capacity.

In soil or substrates nutrient solution is commonly supplied by a drip irrigation system. On one hand it simplifies the supply of the nutrient solution and saves time. On the other hand it needs to be controlled and amounts supplied must be checked to ensure equal supply of nutrient solution that can depend on the quality of the drippers.

Systems without any substrate have the advantage of easy root access by the experimenter. Here, plant roots can be transferred into a nutrient solution that can be:

- circulated as a very thin stream commonly known as a “film,” a nutrient film technique (NFT, Cooper, 1979).
- circulated as a stream, at least 10 mm deep, a deep flow technique or larger container systems (Park et al., 2010).
- frequently sprayed to the roots in so called aeroponic systems (Christie and Nichols, 2004).

If the system is running only with nutrient solution it should be assured that the oxygen concentration in the nutrient solution is sufficient (see Oxygenation of the Nutrient Solution).

### Growing and disposition

Plants have to be assembled in a way that they receive equal radiation. This adjustment determines the plant number per area. The higher the radiation the more plants can be cultivated at the same area. When indeterminate tomato are grown in long-term experiments, no more than 3.5 plants per m^2^ can be grown, at lower light levels this density decreases (see Air Temperature and Radiation). Using other plant types and depending on the growing period until harvest the plant density can be higher.

Plants are not manipulated when a determinate or semi-determinate type is used. Staking and pruning are necessary if an indeterminate type is selected in experiments with a longer cultivation period (about more than 5 weeks). Side shoots on the main stem have to be removed. To have sufficient and balanced fruit setting and yield, particularly during the first 2–3 months, a good balance between vegetative and reproductive growth is necessary. Thus, depending on cultivar type, fruit pruning might be necessary. As a simple rule, the larger the fruits the more the trusses have to be pruned to maintain a balanced growth. The intermediate-type fruited cultivars should have not more than eight and the beef-type not more than six fruits per truss (see Table 1).

During or after transplanting the primary root is usually damaged because of the limited pot size/root environment. A dense lateral root system will develop three levels of branches. In addition aerial or adventitious roots will grow on the stem and must be removed when they disturb or affect the aim of the investigation.

### Environmental requirements—climate

#### General consideration

Tomato can be grown under a wider range of environmental conditions. In most experiments these conditions are artificially controlled in greenhouses or growth chambers. The climate appropriate for an experiment depends on the question under investigation and may also effect the decision for the appropriate place of cultivation. Most topics require optimal growing conditions while some need an unfavorable environment—a specific kind of stress. Unfortunately, the experimental facilities often limit the range of controllable climatic conditions, the experimental area or the fluxes of water and nutrients to the plants. These constraints and the aim of the investigation must be taken into account when planning the climatic conditions for an experiment. An improper climate during the experiment for the question under investigation may lead to a result with accidental artifacts.

One very important premise for adequate climate control in the experimental facilities is the check of the adjusted values of the environmental variables. Aged light bulbs demonstrate a reduced light output from what the manufacture specifies about their product when new. As light bulbs age the emitted light reduces by approximately 20% after 10,000–20,000 h, but also may change the light spectrum. This is very important for growth chambers, especially when more than one chamber is used in an experiment and equal light conditions are required. Producers of greenhouses and growth chambers often do not use very accurate, expensive sensors. In particular several sensors for measuring the CO_2_ concentration are known to demonstrate considerable drift. Also the sensor location is important. In growth chambers, sensors are usually installed in a channel of the climate conditioning system. Depending on the air flow, the temperature and the humidity close to the plant may then differ significantly from the adjusted values. In both growth chambers and greenhouses the light distribution may vary considerably, and the distribution of the temperature may to some extent vary. Light distribution in growth chambers can easily be measured using a hand held light sensor and, if necessary, included in the analysis of the results. In greenhouses this is more complicated, because the light distribution may change with the position of the sun in the sky during the experiment. Therefore, measurements must be simultaneous or repeated during the course of the experiment. In any case, experiments should be conducted in a complete block design.

#### Air temperature and radiation

A necessary premise for any experiment demanding reasonable plant growth is that the temperature must be in line with the radiation. In general, tomato plants grow in a temperature range of 10–35°C and, survive temperatures up to 40°C and down to 0°C. Plants grow already at low daily integrals of 2 mol m^−2^ photosynthetic active radiation (PAR) but also can utilize high photosynthetic photon flux densities (PPFD) of 2000 μmol m^−2^ s^−1^. However, when the temperature does not match the radiation abnormal plant growth can occur. An indeterminate tomato plant develops three leaves per week at a daily average temperature of 20°C on the main stem and, depending on cultivar, between 6 and 8 weeks after flowering fruits will reach the (red) ripe stage (De Koning, 1994; Heuvelink, 1995a). The time for these phenological developments is negatively correlated with the temperature (Adams et al., 2001). The described growth of the single organs demands a certain amount of carbohydrates created from photosynthesis and thus, requires a sufficient daily integral of PPFD. If this is not available, physiological disorders may occur (see Physiological disorders). Plants may become very thin, leaf thickness can dramatically decrease, root growth can be reduced resulting in possible water and nutrient uptake deficiencies that can make plants more sensitive to diseases. In particular, when cultivation aims on fruit or seed production, a higher rate of carbohydrate production is required as compared to plants in their vegetative phase. Carbohydrate deficits result in restricted or suppressed fruit set (Heuvelink, 1995a). Therefore, when daily PAR is limited in the available experimental facility, the low carbohydrate production rate must be brought in line with a low development rate. This is possible in certain constraints by growing the plants at low temperatures.

***Growing young plants in a growth chamber***. Young, vegetative tomato plants may be grown in almost every growth chamber at a PPFD of 300 μmol m^−2^ s^−1^ during a 12–16 h lasting light phase. The resulting daily integral of the PAR of 13.0–17.3 mol m^−2^ d^−1^ is essential for an adequate carbohydrate production and growth at daily average temperatures from 18 to 22°C (Ingestad et al., 1994). These are suitable conditions for many research topics. Either the same temperature during the light and dark phase must be maintained or a higher temperature during the light than the dark phase is possible. A higher temperature during the dark than the light phase should be avoided, because this is unusual in the natural habit and leads to a compact growth (De Koning, 1988; Bertram and Karlsen, 1994). Between the dark and light phases a short dawn and dusk phase may be included but is not essential. The changeover from one temperature set point to another is not instantaneous and is smoothed in growth chambers due to technical restrictions.

Shorter day lengths down to 8 h or lower PPFD down to 150 μmol m^−2^ s^−1^ would be possible but result in plants with thin leaves and low dry matter content (Ingestad et al., 1994). Whether or not the absence of a dark phase results in specific effects is still under debate and therefore a 24 h light phase is not recommendable (Velez-Ramirez et al., 2011).

Also higher PPFD's up to 700 μmol m^−2^ s^−1^ can be applied without damaging the plants. However, at a daily PAR of about 20 mol m^−2^ (e.g., 16 h, 350 μmol m^−2^ s^−1^) the maximum relative growth rate in the exponential growth stage of 0.35 d^−1^ is already reached, that means the plant mass doubles every 2 days at the optimum temperature of 25°C. For comparison, the daily PAR of a completely sunny day in June on the Northern hemisphere is about 70 mol m^−2^. Higher PAR than 20 mol m^−2^ d^−1^ cannot be utilized by young tomato plants in the exponential growth stage, but also not by other species (Ingestad et al., 1994). The exponential growth stage persists until the plant dry mass reaches values of about 2 g. Later, when self-shading of the leaves occurs also higher PPFD's can be utilized by the plants. When a time course of a natural day should be simulated, young tomato plants survive also much higher PPFD's up to 2000 μmol m^−2^ s^−1^ for the corresponding short time under ample water supply without damage. However, such high PPFD's cannot be realized in commercial growth chambers.

***Producing fruits and seeds in a growth chamber***. Generally, cultivating fruit bearing plants requires a higher rate of carbohydrate production and thus, a higher daily PAR than just growing vegetative plants. When indeterminate plants are grown, as in commercial greenhouse tomato production, about 65–75% of the carbohydrates originating from photosynthesis must be distributed to the fruits to enable their growth (Heuvelink, 1995b). Assuming a PAR of 17.3 mol m^−2^ d^−1^ (300 μmol m^−2^ s^−1^, 16 h daily light phase), a light use efficiency of 1 g carbohydrate per mol PAR (Kläring and Krumbein, 2013), 70% carbohydrates allocated to the fruit and a fruit carbohydrate content depending on fruit type of 4–7% arrives at a daily fruit growth of approximately 170–300 g m^−2^. At a temperature of 20°C the time from flowering to the harvest of red ripe fruits is about 8 weeks, and one truss is getting ripe per week (Adams et al., 2001). With three plants per m^2^ in the chamber this yields a possible production of approximately 20 cherry-type, seven intermediate-type or four beef-type tomato fruits per truss (see Background, General use of Tomato for Experimental Purposes), which is about 60% of the typical truss size found in commercial greenhouse production (see Growing and Disposition, Table 1). Increasing the daily PAR is a good possibility to increase fruit production. Decreasing air temperature delays fruit development and thus increases the carbohydrate allocation to the truss due to the prolonged growth phase. This however, is mostly unwanted, because it also prolongs the duration of the experiment. If both, increasing the PAR or decreasing the temperature is not possible then the number of the fruits on the trusses should be manually restricted in order to avoid fruit abortion on the upper flower trusses. Most experiments carried out in growth chambers do not aim on unlimited growth of the stem and plants are stopped after having several flower trusses. Then, carbohydrates are not necessary for vegetative growth anymore and are distributed only to a few growing fruit trusses. Thus, using the determinate type would be another option. In many cases, tomato experiments are possible in growth chambers, even when PPFD's of maximum 300 μmol m^−2^ s^−1^ are available.

***Cultivating plants in the greenhouse***. Other than in a growth chamber, PAR in most greenhouses is limited to the natural conditions and large parts of the solar radiation are absorbed and reflected by the greenhouse cover. While in commercial greenhouses 70–80% of the outside solar radiation reaches the plants, this fraction is much smaller in experimental greenhouses, especially during the winter due to specific equipment and separating walls between small experimental units. Thus, the light transmission coefficient here often reaches only values between 30 and 50%. Therefore, under conditions above the 50°N (degree of Northern latitude) the mean daily PAR from November to January inside the greenhouse is on cloudy days below 2 mol m^−2^ d^−1^, which makes experiments without supplemental artificial lighting very difficult. In particular when experiments focus on fruit production tomato should not be planted under such conditions.

The mean temperature optimal for fruit production is assumed to be 19–20°C (Van der Ploeg and Heuvelink, 2005). However, with increasing outside temperature in the season the temperature in the greenhouse may rise even above 30°C. For the balance between carbohydrate production, growth, and phenological development this is not a problem, because the high temperatures are associated with high solar radiation. High temperature is also a concern with experiments in a hot climate. However, temperatures above 33°C must be avoided with most cultivars when aiming to produce fruit because pollen formation is hindered and pollen viability is reduced (Adams et al., 2001; Domínguez et al., 2005). Using a shading system which is installed outside the greenhouse may slightly decrease the temperature in the greenhouse while a screen inside the greenhouse reduces transpiration (see Water Supply) but does not reduce the temperature (Kläring and Krumbein, 2013). However, at outside temperatures above 30°C a healthy, fully developed crop with ample water supply considerably cools the greenhouse air by transpiration and the temperature at crop level may even be less than the outside temperature.

In countries with arid climates so called wet-pads (or pad and fan system) are used to control temperature and relative humidity by evaporative cooling: dry outside air is drawn into a greenhouse through continuously watered pads (Abdel-Ghany and Kozai, 2006). Due to the constraints originating from very high outside temperature and solar radiation experiments with tomato during the summer are problematic in arid areas. Also, in the humid tropics there are almost no possibilities to control the temperature in the greenhouse.

Many experimental (and several commercial) greenhouses are equipped with systems for artificial lighting, usually lamps supplying PPFD's in the range from 100 to 200 μmol m^−2^ s^−1^, which allows using them for experiments year-round even for fruit production (Verheul et al., 2012). A further possibility to (little) compensate for low daily PARs is decreasing the plant density. In particular when cultivating young plants, the final leaf area is often underestimated resulting in an unsuitable high plant density.

If, besides artificial lighting, the greenhouse is equipped with an electrical cooling device, humidification, dehumidification, and (outside) shading, it can be operated more or less like a growth chamber and the rules for climate control of growth chambers can be adapted.

#### Spectrum of radiation

There are many investigations on the effects of specific spectra on growth and especially on fruit quality aspects in tomato. Except for the fractions of the UVA- and UVB-radiation which are significantly reduced by the cover, plants in greenhouses receive full sunlight spectrum. Nowadays there are also UVB-transmissible coverings available. The fact, that UV radiation leads to a somewhat more compact growth (Ballare et al., 1995) is likely unimportant for most experiments as long as all treatments and experiments of a series are conducted under the same conditions. This also concerns additional artificial lighting. As the sunlight provides the entire spectrum necessary for tomato growth and phenological development, for experiments in greenhouses the lamp type is rather unimportant. Therefore, greenhouse lamps were developed to maximize PAR and photosynthesis per Watt of electrical energy consumption. The current standard in greenhouses is the use of high pressure sodium (HPS) discharge lamps. It is expected for the future, that light emitting diodes (LED) with higher energy use efficiency will be available (Van Ieperen, 2012).

The situation in growth chambers is different. Here, sole lamps must provide the entire spectrum necessary for tomato growth. If the growth chamber was supplied by a specialized commercial provider, this should be the case. Nowadays there are lamps available with a spectrum close to the spectrum of sunlight. If fluorescence tubes or HPS lamps are used, it may be essential to have additional lamps that provide radiation in the red and far-red spectrum such as incandescent lamps or LED's (Hogewoning et al., 2010).

#### Relative humidity

In general, a relative humidity of the air between 65 and 75% is optimal for growth, flowering, fruit set, and fruit growth of tomato plants (Bakker, 1991a). These conditions can be easily realized in any growth chamber and experimental greenhouse equipped with technical cooling, humidification, and dehumidification.

It is more complicated in greenhouses with greenhouse specific climate control. In the heating season with cold outside temperatures the relative humidity in the greenhouse drops to low values due to the condensation of water vapor on the colder cover and to the air exchange with the dry outside air. Also, early in the season relative humidity is often very low when only a few or small plants are present in the greenhouse. However, most experimental greenhouses are equipped with a fogging system which enables control of the relative humidity in that situation. Unfortunately, if outside temperature and solar radiation increase, ventilation is required. When greenhouse vents are fully opened the effect of the fogging system on the relative humidity is very limited. In countries with arid climate wet-pads are used to increase relative humidity and to decrease air temperature (see Cultivating Plants in the Greenhouse). Below 30% relative humidity tomato plants still grow but not optimally. Particularly in combination with high temperature, the morphology and physiology is changed: Plant dry matter content is increased and specific leaf area is decreased. Photosynthesis may be decreased due to stomata closure at very high temperatures and low relative humidity (Bakker, 1991b). A decreasing ratio of nutrient to water uptake must be taken into account when controlling water and nutrient supply (see Nutrition Control Strategies). When aiming on fruit production, physiological disorders such as blossom end rot may become a serious problem. Low relative humidity in combination with high temperature accelerates the propagation of harmful insects such as *Trialeurodes vaporariorum* and *Tetranychidaes* but not that of their antagonists such as *Encarsia formosa* and *Gamasinas*. Vice versa, high relative humidity in the greenhouse may occur at high outside temperature and relative humidity, mainly during the night, when the ventilation remains closed because the experimental design requires high daytime temperature or in the humid tropics. As at very low relative humidity, vegetative growth is not inhibited but the ratio of nutrient to water uptake increases with implication for plant nutrition (see Nutrients and Recipes). For fruit production, however, long lasting relative humidity above 85% in combination with high temperature greater than 30°C are critical for fruit set, because pollen can agglutinate and pollination is hindered which may then lead to parthenocarpic fruits (Adams et al., 2001; Domínguez et al., 2005). In addition, high relative humidity favors the incidence of fungal leaf diseases such as powdery mildew. In most situations high relative humidity can be avoided by an appropriate climate control, that means, by ventilating the greenhouse, and, if necessary, heating at the same time.

#### Carbon dioxide concentration

The effect of the carbon dioxide (CO_2_) concentration on plant growth is quite simple and straight forward. With increasing CO_2_ concentration of the air photosynthesis and thus carbohydrate production is enhanced. Raising the CO_2_ concentration from the current ambient level of approx. 400 to 1000 μmol mol^−1^ increases photosynthesis of tomato by approx. 30% (Nederhoff, 1994). This may be used to partly compensate for a lack of radiation, mainly in growth chambers. In greenhouses, however, with raising solar radiation and outside temperature ventilation opens and then high CO_2_ concentrations cannot be kept.

One problem concerning the CO_2_ concentration is often overlooked, especially when a measurement of the CO_2_ concentration is not available: The air exchange rates of growth chambers and (closed) greenhouses are usually below one, what means that in 1 h the air in the facilities is not completely replaced by ambient air. Then, depending on plant density and radiation, the CO_2_ concentration may drop dramatically as CO_2_ demanded is not replenished from outside. In greenhouses, values down to 150 μmol mol^−1^ may occur (Kläring et al., 2007), in growth chambers even below 100 μmol mol^−1^ which is already close to the CO_2_ compensation point of photosynthesis. Increasing the air exchange rate of the growth chamber by pumping outside air into the chamber is one option which is however limited, because, depending on chamber type, at higher air exchange rates the climate control device may not work properly. In greenhouses, opening the ventilation earlier in order to increase the air exchange rate results in a (mainly unwanted) temperature drop which cannot be compensated by heating (Stanghellini et al., 2008). A reduction of plant density would help but usually conflicts with the number of planned treatments or necessary replications. Even if there is no solution, one should be aware of the problem and at least monitor the CO_2_ concentration in the plant's environment.

#### Climate control strategies

Table 5 gives examples for suitable climate control strategies for growing tomato in growth chambers and in the greenhouse. In the growth chambers the adjusted values for the climate variables are realized by the conditioning systems. In a greenhouse without technical cooling the set points for the climate control are constraints. Temperature falling or exceeding a given set point activates a corresponding conditioner but this does not guaranty the deviation control in any circumstances. For example, a target range of 20–22°C in the greenhouse can be controlled by adjusting the set point for heating and ventilating at 20 and 22°C, respectively. At an outside temperature of 30°C, however, the inside temperature in the greenhouse is far from the set point temperature.

**Table 5:** **Examples of set points for climate control in growth chambers and greenhouses in experiments with tomato**.

**Facility**	**Light phase**	**Temperature**	**Relative humidity**	**CO_2_ concentration**	**Water consumption**
Growth chamber	16 h	20°C day and night	70%	400 μmol mol^−1^	Maximum
300 μmol m^−2^ s^−1^	17.3 mol m^−2^ d^−1^	22/18°C day/night			3 l m^−2^ d^−1^
Growth chamber	12–16 h	25°C day and night	75%	400 μmol mol^−1^	Maximum
700 μmol m^−2^ s^−1^	30–40 mol m^−2^ d^−1^	27/23°C day/night			6 l m^−2^ d^−1^
Greenhouse	8–16 h (50°N)	Heating^a^ 19/17°C day/night	Fogging^a^ 60%	Supply^a^	Maximum
					2/6 l m^−2^ d^−1^
No artificial lighting	Winter/summer	Ventilating^a^ 22°C	Ventilating^a^ 80%	400 μmol mol^−1^	Winter/summer

a *Set point of the corresponding controller if available*.

The first strategy is based on a growth chamber with a strong limited light source. Nevertheless, those chambers are suitable for conducting experiments on tomato even for fruit production. Under these circumstances the light phase should reach 16 h and the temperature should be moderate in order to bring in line the phenological development with the dry mass production (see Air Temperature and Radiation).

When a more intensive light source is available, higher temperatures may be adjusted which, thereby are shortening the duration of the experiments. Higher temperatures are still possible if the question under investigation requires it. With increasing temperature also the relative humidity should be slightly increased in order to avoid very high vapor pressure deficits (VPD). In addition, the high water demand of the plants under those conditions should be kept in mind.

In heated greenhouses set points for climate control can be adjusted almost independently of the season (Table 5). The realized greenhouse climate however, is modulated by the outside meteorological variables. Thus, during the winter in a temperate zone the two set points for heating of 19°C by day and 17°C at night result in an average temperature of approximately 18°C. During early spring and late autumn, the duration of the daytime is already longer and the solar radiation is significantly heating the greenhouse arriving at average temperatures in the greenhouse of 19–21°C. When the outside temperature exceeds the set point for ventilation in spring, summer, and autumn, daily average temperature also increases and may reach values up to 25°C in the greenhouse on hot summer days. On lower latitudes with higher temperatures and solar radiation the realized average temperatures in the greenhouse are accordingly higher.

### Nutritional requirements

#### Water supply

High quality water is a prerequisite for irrigation in experiments (Peet, 2005). Several water sources are generally available but depending on the research facility water resources may be limited: deionized water (safest choice), rainwater, tap or well water, and surface water from lakes or other sources (worst choice). The water quality of the different sources varies tremendously because of its ion concentrations and contaminations with biological agents (Table 6; Schwarz et al., 2004). Therefore, some sources might not be suitable for irrigation since their quality reaches characteristics above thresholds (Sonneveld et al., 1991; Wetzel, 2001).

**Table 6:** **Chemical composition of different potential water sources (modified after Wetzel, 2001; Sonneveld et al., 1991; Schwarz et al., 2004)**.

	**Rainwater**	**Tap-, well-water**	**Surface water**	**Thresholds**
EC^a^, dS m^−1^	<0.1	0.1–2.2	>0.3	0.5
pH	4–6	7–8.8		
**ION/ELEMENT, μmol l^−1^**				
Na^+^	9–50	50–250	>90	
Cl^−^			>10	
NO^−^_3_	30–100	0.1–20	>50	>^b^
NH^+^_4_	7–36			>
K^+^	2–13	2.5–750		>
Ca^2+^	5–100	2.5–4200	>750	2000
Mg^2+^	2–21	4–2100	>60	500
SO^2−^_4_	30–100	3–5000	>60	500
HCO^−^_3_		0.3–100	>450	10000

a EC, electrical conductivity;

b *no thresholds, typically lower than required by plants*.

Preferentially, deionized water should be used since it does not carry any additional nutrients or unwanted ions into the substrate and thus to the plants. However, application without any ions may easily result in plant damage because of ion removal from plant cells. Deionized water has only low concentrations of microorganisms. If not available, rainwater collection, and its use is an alternative. However, sometimes it may contain harmful microorganisms such as *Pythium aphanidermatum*. Pathogen contamination can also be found in surface water sources and both sources may need disinfection. If the facility is equipped with a disinfection system then rain water will be a good alternative to deionized water.

Tap water is the water source mostly applied. However, users sometimes do not consider that tap water contains already quite high concentrations of divalent anions, sodium and chloride (see Table 6) and, that these concentrations may vary with time due to changing sources in the supply chain. This might result in decreased plant growth and even toxification of the tomato. When used, in all cases the ion composition of tap water and water from surface sources should be tested and known.

The water demand is determined by transpiration, evaporation, and the amount of water remaining in the plant, whereby the transpiration is the main fraction. The fraction embedded in the increasing biomass is quite low: Based on a water use efficiency of 5 kg dry mass per 1 m^2^ water taken up by the plants (Kläring and Krumbein, 2013), and a water content of 95% in the fruit and 85–90% in the vegetative biomass, a fraction of the total water uptake of approximately 10% is remaining in the fruit and only 3–5% is remaining in the vegetative biomass. Also, the evaporation can be neglected if plants are not grown in beds due to the relatively small surface area of the growing medium compared to the transpiring leaf area. Moreover, evaporation can be minimized by covering the vessels, containers etc. where the plants grow with a foil. Thus, the water supply to the plants must primarily compensate for the plant's transpiration, and then have a secondary purpose to flush the growing medium in order to avoid ion imbalances (see Nutrients and Recipes, Physiological Disorders Caused by Climate, or Fertigation Mismanagement). Water logging must be avoided because this leads to hypoxia and anoxia in the root environment (see Oxygenation of the Nutrient Solution). Transpiration of tomato plants and thus, water demand is similar to that of most cultivated herbaceous plants. In a wide range of climatic conditions it increases with increasing leaf area, radiation and VPD of the air.

For simple pot experiments it is very common not to follow the transpiration demands directly but to maintain a certain water content in the growing medium, considering its water holding capacity. Based on these characteristics, the growing medium can be watered until a wanted proportion of the field capacity is reached (e.g., 85%), and it can be maintained during the experiment using a balance. Experimenter has to take care that the water content does not fall below the permanent wilting point.

Automated fertigation systems combining water and nutrient supply are available and reduce the time for watering immensely. They can be used in pot/container experiments as well as in in long-term experiments carried out in commercial cultivation systems with or without a growing medium. In these systems dripping time and frequency depend on leaf area and radiation. It must be taken into account that both the supply rate of the drippers and the size of the plants, thus their transpiration rate, underlie a variance. Therefore, a surplus of 30–50% of the mean water uptake by the plants should be applied.

In many experiments however, the size of the plants is very different due to the treatments which also require different water supply rates in order to avoid different moistures of the growing medium between the treatments. Sometimes, different water qualities for the treatments are required. Thus, in many experiments fully automated irrigation is not possible.

Aiming on a large number of treatments and replications in a compact experimental design, not only the final size of the plants and their corresponding transpiration are often underestimated but also the actual field capacity of the substrate is frequently overestimated. If irrigation is not automated this leads to measures such as manually watering by hand one or more times per day, shading the plants which results in arbitrary low ratios of PAR to temperature or, in the worst case scenario causing water deficits and thus, resulting in artifacts.

In most cases the leaf area index by the end of the experiment is 2 or larger, that is, two units of leaf area are grown per one unit of ground area. Then the transpiration of tomato plants in a greenhouse during the summer as well as under full illumination in a growth chamber may reach values of 6 l m^−2^ d^−1^, independently of whether the space is occupied by many small or few adult plants. Here constraints may arise, especially when substrates with a low available water capacity such as gravel are used (see Growing Systems and Growing Media, Table 4). For example, in an experiment with plants grown in sandy soil with an available water capacity of 0.1 l l^−1^ at a density of 10 plants per m^2^ the minimum root zone volume must be greater than 6 l in order to avoid plant water deficit at one water supply rate per day. In addition, the variation of the plant size has to be taken into account, which means, that some plants require more than 0.6 l d^−1^.

Beside the automation of the irrigation, preferably using a combined time and light-integral controlled water supply, the climate may be adapted. This, however, should be decided earlier when designing the experiment. In growth chambers this is easy to achieve. As transpiration is affected mainly by radiation and VPD of the air, growing plants at lower PPFD's, and lower temperature may be a reasonable compromise which significantly reduces the transpiration rates of the plants but also prolongs the duration of the experiment (see Producing Fruits and Seeds in a Growth Chamber). In the greenhouse without technical cooling there are much more constraints. Automatically shading the plants at high solar radiation intensities is one option. However, it has a limited impact because it reduces photosynthesis and dry mass growth in the same order of magnitude as transpiration (Kläring and Krumbein, 2013). Thus, in greenhouses remain three possibilities to overcome the trouble: automation of the water supply—the best solution, choosing a suitable container size and (if question under investigation allows) a growing medium with a high effective field capacity/water volume and, shifting the experiments into another time of the year—probably the worst solution.

#### Nutrients and recipes

If this is not a matter of investigation not only water but also all plant nutrients should be provided in an optimal manner and the supply of unwanted ions, such as sodium, chloride, or heavy metals should be eliminated. Some water sources, such as tap water, (see Water Supply, Table 6) contain already high amounts of calcium, magnesium, or sulfate in concentrations that can be sufficient to provide tomato with enough of these nutrients. Also sodium and chloride can be present and accumulate in the irrigation system.

For container experiments with a growing medium and pure water supply (no nutrients), the medium should be fertilized with a basic amount of nutrients that provides the tomato with enough nutrients for the growth period. Although tomato is a salt tolerant plant, salinization of the growing medium must be avoided. Thus, as a rule, all nutrients necessary for a long-term experiment cannot be applied already from the beginning but must be added continuously and at a later stage of plant development. Table 7.1 offers an example for nutrient supply in a pot experiment calculated per liter growing medium and related to a tomato plant of maximum 200 g fresh mass. However, values are only for orientation and cannot be generalized for all conditions and growing media because of the reasons mentioned before.

Table 7**Nutrient and fertilizer supply**.

**Table:** 

**7.1. Example for nutrient and fertilizer supply (or lab chemical) to a pot experiment where coarse sand was used as a substrate**			
**Element/nutrient**	**g kg^−1^ substrate**	**Fertilizer/chemical**	**Per kg substrate**
N	1.5	Ca(NO_3_)_2_ (g)	12.6
P	0.2	CaSO_4_ (g)	4.0
K	1.5	KH_2_PO_4_ (g)	0.88
Ca	0.7	K_2_SO_4_ (g)	2.9
Mg	0.2	K_2_O-MgO (g)	2.0
S	0.4		
Fe	0.007	Fe-EDTTA (ml)	0.1
Mn	0.006	MnSO_4_ (mg)	18.0
Zn	0.008	ZnSO_4_ (mg)	20.0
B	0.01	H_3_BO_3_ (mg)	57.0
Cu	0.008	CuSO_4_ (mg)	32.0
Mo	0.001	MoO_4_ (mg)	1.7

**Table:** 

**7.2. Nutrient solution recipe for tomato (De Kreij et al., 1997)**			
**Nutrient/element**	**Starter solution**	**Root environment (range)**	**Refilling solution**
EC^*^ in dS m^−1^	3.7	2.5–5.5	1.5
pH	5.6	5–6	5.6
**mmol l^−1^**			
NO_3_	23	15–31	10.75
NH_4_	0.1	0.1–0.5	1.0
K	8	5.3–10.6	6.5
Ca	10	6.6–13.3	2.75
Mg	4.5	3–6	1.0
SO_4_	6.8	4.5–9.0	1.5
P	1.0	0.7–1.3	1.25
HCO_3_	<1	0–1.0	
Na	<12	0.1–12.0	0
Cl	<15	0.1–15.0	0
**μmol l^−1^**			
Fe	25	13–38	15
Mn	5	2–7.5	10
Zn	7	3.5–10.5	4
B	50	25–75	20
Cu	0.75	0.4–1.1	0.75
Mo	0.5	0.3–0.8	0.5

**Table:** 

**7.3. Changes of the nutrient solution during different phenological growing stages (De Kreij et al., 1997 simplified)**									
**Stage**	**Adaptation, mmol/l**								
	**NH_4_**	**K**	**Ca**	**Mg**	**NO_3_**	**SO_4_**	**H_3_PO_4_**	**B^a^**	**Fe^a^**
Start	−1^b^	−3.5	−1.25	+1^c^	−1.5	+1	−0.5	+10	+10
Flower first truss		−1.2	+0.3	+0.3					
Flower third truss		+1.0	−0.25	−0.25					
Flower fifth truss		+3.5	−1.25	−0.5					
Flower tenth truss		+1.0	−0.25	−0.25					

* *EC, electrical conductivity*.

a in μmol/l;

b “−” reduce;

c *“+” increase compared with the standard (see Table 7.2)*.

The concentration of the macro nutrients should be regularly monitored in the growing medium used in the experiment. A small amount of the growing medium sampled and analyzed in a specialized lab is important to check if plant demands are fulfilled. It is easier to place a sensor in the drain solution with a simple measurement of its electrical conductivity (EC). However, the EC indicates only the total amount of soluble ions available for uptake. Mainly macro-nutrients but no micro-nutrients are considered since their contribution to the total amounts of soluble ions is rather small. Roughly characterized, values below 1 dS m^−1^ in the drain signify not enough nutrients for uptake and above 7 dS m^−1^ a danger of salinization.

For experiments with nutrient solutions a large number of recipes are available that have been developed by many research teams at different locations worldwide. The most famous recipe is the so called Hoagland (1944) improved by Hoagland and Arnon (1950), a universal recipe, still used by researchers for many purposes and plants. However, all of these recipes were identified empirically and have their limits since they do not follow the exact demands of a tomato crop. The most tested, investigated and applied recipe is that of a Dutch team (De Kreij et al., 1997). They evaluated and optimized a composition of a nutrient solution that is adapted to the growing conditions, medium used, and the developmental stage of the tomato. The reason for the adaptations is that the content of the single elements differ from plant organ to organ. The calcium content for example, is between 2 and 5% in the leaf dry mass but only 0.15% in the fruit dry mass. Consequently, the fraction of calcium ions in the nutrient solution supplied must be higher in the vegetative growth phase than in the fruit growth phase. Therefore, recommendations begin with a starter recipe to satisfy the tomato in the root environment and to ensure optimal uptake (Table 7.2). Thereafter, a change to a second recipe is necessary that follows plant demands in uptake. Moreover, it has several adaptations considering different phenological plant stages, such as flowering of the first, fifth and tenth truss (Table 7.3).

#### Nutrition control strategies

**Figure 1:**
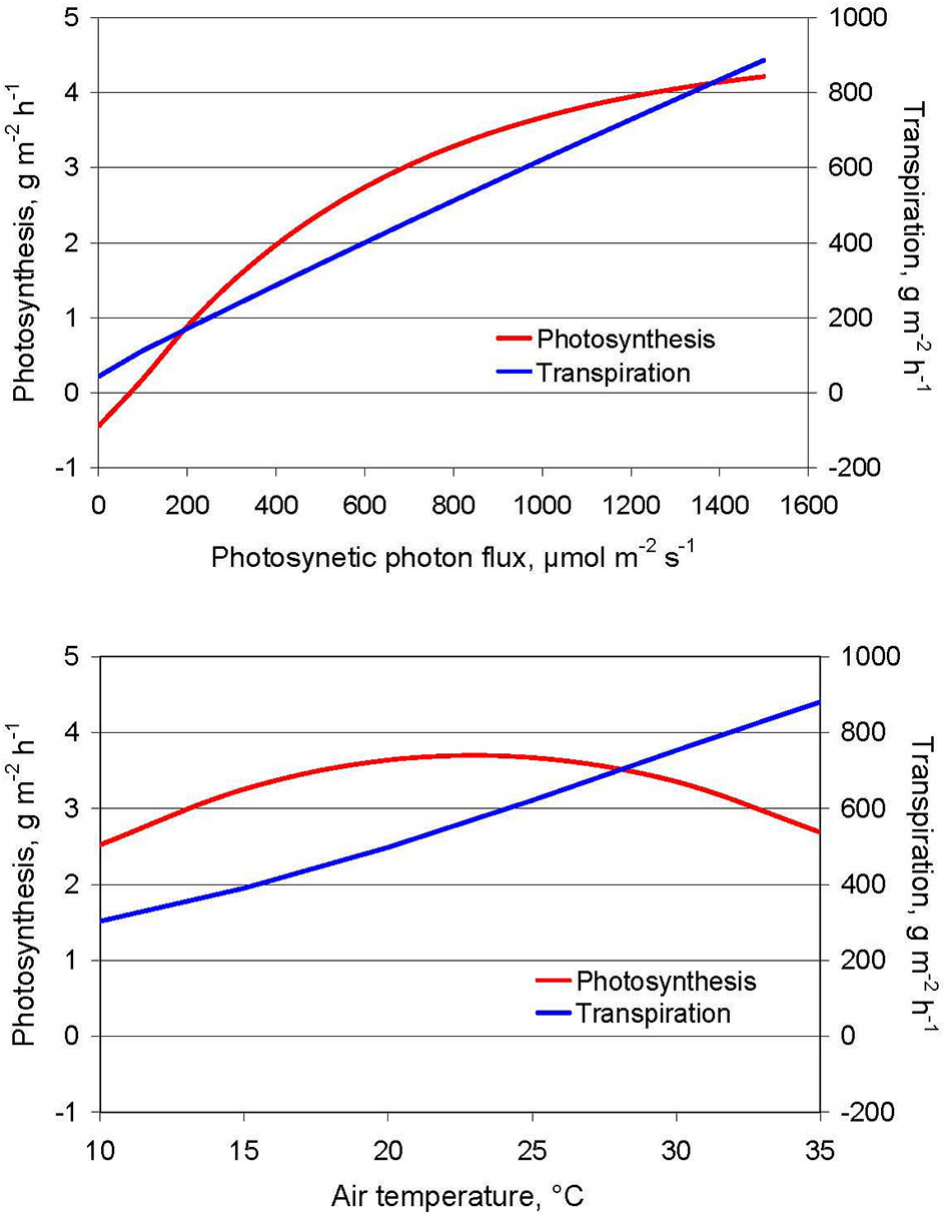
**Effect of photosynthetic photon flux at 25°C (above) and air temperature at 1000 μmol m^−2^ s^−1^ PPFD (below) on tomato crop photosynthesis and transpiration under unlimited water supply**. Relative humidity of the air is always 70%, thus vapor pressure deficit is decreasing with temperature. Data are derived using a crop growth model (Kläring and Bar-Yosef, 2006).

The environmental conditions affect mainly the ratio of nutrient to water uptake. Nutrient uptake must ensure the growth in plant biomass which relies on the carbon assimilation in the photosynthesis. Water uptake is related to transpiration. With increasing radiation both photosynthesis and transpiration increase and their ratio remains almost unchanged. The effect of temperature on photosynthesis is very low, while it has a strong impact on transpiration (Figure 1). This significantly changes the ratio of nutrient to water uptake of the plants. Changing nutrient/water uptake ratios do not appear in growth chambers with a constant climate but may become an important error source in greenhouse experiments. In particular on hot summer days, temperature in the greenhouse may considerably rise, and relative humidity decrease. As a result, very high transpiration rates occur and plant's water uptake markedly increases, also in relation to photosynthesis and nutrient uptake (Kläring et al., 1997; Schwarz et al., 2001). This will result in very high total ion concentration and thus in unwanted changes of the osmotic conditions in the root environment with many disrupting side effects. Vice versa, very low VPD over a longer period may result in nutrient depletion in the solution due to the low water uptake in relation to nutrient uptake.

When supplying nutrient solutions to plants this should be taken into account by increasing or decreasing the nutrient concentration in the nutrient solution at decreasing or increasing VPD, respectively. When larger amounts of nutrient solution are required, usually high concentrated stock solutions are prepared. They are then diluted until the appropriate nutrient to water ratio is reached. This ratio is controlled by adjusting the necessary value of the EC of the nutrient solution. The recommended EC as well as the optimal pH is component of the recipe. As the EC accounts for all ions in the solution the effect of ballast ions, such as sodium and chloride, stemming from the fresh water must be taken into account (see Water Supply) as well as the above described effect of the climate.

#### Oxygenation of the nutrient solution

Tomato plants are very sensitive to oxygen deprivation (hypoxia) in the root environment. The symptoms of hypoxia such as wilting become temporarily visible when plants are irreversibly damaged. In water culture, adult tomato plants fade away within 2 days at oxygen concentration less than 3 mg l^−1^. Already a few hours of hypoxia in the root environment results in a serious damage of the roots accompanied by a marked growth reduction although symptoms are not visible. Young plants may adapt to hypoxia in the root environment but also respond with irregular growth (Kläring and Zude, 2009). Therefore, adequate oxygen concentration in the root environment is essential for any experiment. When plants are grown in containers with nutrient solution, the solution must be aerated and the oxygen concentration should be above 4 mg l^−1^. In systems where nutrient solution is permanently flowing along the roots the solution must be aerated. In systems with a pulsed flow which result in a very thin moisture film on the roots gullies should have a gradient between 1 and 2%. Water pockets must be absolutely avoided, due to the fact that hypoxia can occur causing root death that can then lead to a favorable environment for root diseases. Infection would eventually expand over the entire system. In containers and slaps filled with rockwool or other substrates, water logging must be avoided. In almost all substrates this does not occur as long as there is a free drainage close to the bottom.

#### Physiological disorders caused by climate or Fertigation Mismanagement

Tomato can show a variety of nutrient deficiencies, with symptoms that can be element, cultivar, or condition specific. Concentrations higher than the optimum may occur as well, especially for micronutrients. Non-optimal nutrient supply is often accompanied or even forced by unfavorable climate conditions. An overview and detailed description of the many disorders can be found in Peet (2005, 2009). Among the many potential deficiencies that can occur “Blossom End Rot” is often found and may lead to a breakdown of the whole experiment. It appears as a brownish dry and firm sunken area at the blossom end of the fruits. It is obviously caused by several reasons, such as low calcium availability, limited uptake and transport, high salinity or ammonium supply, low or very high humidity, uneven watering, high growth rates. Therefore, balanced fertilization and common growing conditions (no extremes) help to prevent this disorder. Another symptom related to nutrient disorders (low K, high N) is “Blotchy Ripening.” Symptoms, such as “Cracking,” and “Russeting,” are related to an uneven water supply. “Catfacing,” “Misshapen fruit,” “Puffiness,” “Sunscald,” “Green or Yellow Shoulders” are related to unfavorable climate conditions.

### Conflict of interest statement

The authors declare that the research was conducted in the absence of any commercial or financial relationships that could be construed as a potential conflict of interest.

## Footnotes

^1^Tomato Genetic Ressource Center (TGRC) at the University of California, Davis, USA. http://tgrc.ucdavis.edu
